# Sucrose Treatment Enhances the Electrotransfer of DNA by Activating Phospholipase A2

**DOI:** 10.3390/pharmaceutics16040475

**Published:** 2024-03-29

**Authors:** Chunxi Wang, Chun-Chi Chang, Jen-Tsan Chi, Fan Yuan

**Affiliations:** 1 Department of Biomedical Engineering, Duke University, Durham, NC 27708, USA; 2 Department of Molecular Genetics and Microbiology, Duke University School of Medicine, Durham, NC 27710, USA

**Keywords:** sucrose, phospholipase A2, V-ATPase, vesicle trafficking, gene electrotransfer

## Abstract

Our previous study discovered that sucrose and other non-reducing sugars (e.g., trehalose and raffinose) could be used to improve the electrotransfer (ET) of molecular cargo, including DNA, mRNA, and ribonucleoprotein in various cell lines and primary human cells in vitro and in vivo. To understand the molecular mechanisms of this improvement, we used RNA sequencing technology to analyze changes in the cell transcriptome after sucrose treatment. The results from our analysis demonstrated that the sucrose treatment upregulated phospholipase A2 and V-ATPase gene families, which could potentially influence the acidity of intracellular vesicles through augmenting vesicle fusion and the influx of proton, respectively. To determine how this upregulation affects ET efficiency, we treated cells with pharmaceutical inhibitors of phospholipase A2 and V-ATPase. The data demonstrated that the treatment with the phospholipase A2 inhibitor could reverse the ET improvement elicited by the sucrose treatment. The V-ATPase inhibitor treatment either had little influence or further enhanced the effect of the sucrose treatment on the ET efficiency. These observations provide a molecular explanation for our previous findings, demonstrating that the sucrose treatment primarily enhanced the ET efficiency by promoting vesicle trafficking and fusion through the activation of phospholipase A2.

## 1. Introduction

Sucrose is a disaccharide composed of glucose and fructose. It is widely known as table sugar and plays essential roles in our daily life. Besides its use as a sweetener, sucrose also finds numerous applications in biomedical sciences. Sucrose has been commonly used as a stabilizing agent in the preservation of biological samples, such as proteins, extracellular vesicles, cells, and tissues, during cryopreservation [1,2,3,4]. Its ability to prevent the formation of ice crystals and structural damage has led to its incorporation in the formulation of cryoprotectants used in storing cells and tissues for transplantation and in the preservation of virus-based and lipid nanoparticle (LNP)-formulated vaccines [5,6,7,8]. For example, sucrose is a listed ingredient in the mRNA-LNP vaccines for COVID-19 provided by both Pfizer-BioNTech and Moderna that helps maintain the stability of the vaccine after its production [9,10]. Additionally, sucrose is used in density gradient centrifugation to isolate specific cellular components, such as exosomes and Golgi membranes, based on their buoyant density [11,12]. Despite its significance in cell-, tissue-, and vaccine-related biopharmaceutical and research applications, the impact of sucrose exposure on cell and tissue physiology has rarely been explored. Understanding such impact is important for broadening the use of sucrose in clinical practices and understanding the mechanisms of findings in sucrose-related applications.

Sucrose has also been used in drug and gene delivery [13,14,15]. One of its applications is in improving electrotransfer (ET) [14,16,17], which is a technology for the delivery of molecular cargo, including DNA, RNA, protein, and ribonucleoprotein (RNP) [18,19,20]. ET is cost-effective, versatile, multiplexable, and applicable to almost all cell types [21,22]. It is usually performed with the help of specially formulated buffers to achieve optimal cell viability and delivery efficiency [23]. Sucrose has been used as an important component in ET buffers as an osmotic balancing agent [14,16,17]. Recently, we discovered that ET efficiency could be enhanced through the pretreatment of cells with sucrose in vitro [24], partly due to the formation of large, amphisome-like bodies (ALBs) and enlargement of lysosomes. This enhancement has been observed in tumor cell lines (e.g., HCT116, HT29, and B16.F10) and non-tumor cell lines (e.g., C2C12, DC2.4, and HEK293), as well as in primary human cells (e.g., dendritic cells and T cells isolated from peripheral blood mononuclear cells) [24,25,26]. To extend the applications of sucrose in vivo, we showed that sucrose encapsulated in LNPs could be used as a non-inflammatory adjuvant to DNA vaccines by improving the efficiency of ET in mice [26]. In this previous study, we also demonstrated, in vivo, that to achieve the same enhancement in transgene expression post ET, the required sucrose treatment was equivalent to increasing the dose of DNA approximately 3000-fold. Mechanistically, the sucrose treatment improves ET efficiency by reducing the lysosomal acidity that impairs the capability of lysosomes to degrade exogenous DNA delivered into cells, leading to prolonged transgene expression [24,26]. However, the detailed molecular mechanisms behind how the sucrose treatment is able to alter intracellular vesicle functions and vesicle–vesicle interactions are still unknown. To understand these mechanisms, in the present study, we investigated transcriptional responses to the sucrose treatment and determined how changes in endogenous gene expression affected ET efficiency.

## 2. Materials and Methods

### 2.1. Cell Preparation

HCT116 cells were purchased from ATCC. They were cultured in McCoy’s 5A medium (Gibco, Grand Island, NY, USA) supplemented with 10% bovine calf serum (BCS) and 1% Pen-Strep (P/S, Gibco). Cells were maintained at 37 °C in a humidified incubator with 5% CO_2_. For the subculture, cells were detached from flasks with a 0.25% (*w*/*v*) Trypsin–0.53 mM EDTA solution. The cell culture medium was renewed every two to three days.

### 2.2. Plasmid Preparation

DNA plasmid encoding the enhanced green fluorescent protein (EGFP) was purchased from Nova Lifetech Inc. (Hong Kong, China) (pEGFP-N1) and prepared using the PureLink Quick Plasmid Miniprep Kit (ThermoFisher Scientific, Waltham, MA, USA). The concentration of the plasmid was quantified using a NanoDrop One Spectrophotometer (ThermoFisher Scientific, Waltham, MA, USA). The plasmid was diluted in PBS (Gibco) to the desired concentrations before use.

### 2.3. Electrotransfer of Plasmid

Cells were prepared for ET as previously described [21]. Briefly, cells were collected and suspended in Opti-MEM™ Reduced Serum Medium, GlutaMAXTM Supplement (Gibco), at a concentration of 10 × 10^6^/mL. After this, the plasmid DNA was added to the suspension at a concentration of 10 μg/mL. For each sample, a 100 μL suspension was transferred to a cuvette with aluminum electrodes and a 4 mm gap (Bio-Rad, Hercules, CA, USA). Two pulses of 250 V in field strength, 10 ms in duration, and 0.1 Hz in frequency were delivered to the cells using the BTX ECM 830 Square Wave Electroporation System (Harvard Apparatus, Holliston, MA, USA). Cells were immediately transferred to prewarmed cell culture media after ET, and cultured at 37 °C in a humidified incubator with 5% CO_2_.

### 2.4. Inhibitor Treatment

Oleyloxyethyl Phosphorylcholine and Concanamycin A were purchased from Santa Cruz Biotechnology. The Oleyloxyethyl Phosphorylcholine was initially dissolved in ethanol, and then diluted in cell culture medium before being added onto the cells. The Concanamycin A was initially dissolved in DMSO and then diluted in cell culture medium before being added onto the cells. Prior to the inhibitor treatment, HCT116 cells were cultured in medium containing 100 mM sucrose or medium alone (no treatment) for 24 h. Then, the medium was aspirated and replaced with fresh medium containing different concentrations of inhibitors, and the cells were further incubated for 6 h before being harvested for ET.

### 2.5. RNA Sequencing and Pathway Analysis

HCT116 cells were cultured in the medium without or with sucrose (100 mM) for 24 h. The cell samples were then collected for total RNA extraction with a Qiagen RNeasy kit. For each treatment group, there were three biological repeats. All RNA samples were submitted to the Genomic and Computational Biology (GCB) core facility at Duke University for library preparation using KAPA Stranded mRNA-Seq Kit (Kapa Biosystems, Wilmington, MA, USA). The libraries were sequenced on one lane of HiSeq 4000, and the RNA-seq data were processed using the TrimGalore toolkit, which employs Cutadapt to trim low-quality bases and Illumina sequencing adapters from the 3′ end of the reads. The reads that were 20 nt or longer after trimming were mapped to the GRCh37v75 version of the human genome and transcriptome using the STAR RNA-seq alignment tool. For the reads that could be mapped to single genomic locations, gene counts were compiled using the HTSeq tool; only genes that had at least 10 reads in any given library were used in subsequent analyses. The raw count data were used for differential expression analysis using the DESeq2 Bioconductor package with the R statistical programming environment. The false discovery rate (FDR) was calculated to control for multiple-hypothesis testing.

Gene set enrichment analysis (GSEA) was performed with the GSEA software (GSEA_4.3.2) and C5 gene sets [27,28]. The gene set filters (min = 15, max = 500) were employed to eliminate smaller and larger sets. Consequently, only 7604 out of 16008 gene sets were used for the identification of differentially regulated pathways and Gene Ontology (GO) terms for each of the comparisons performed. Outcomes of the analysis were verified with the fold enrichment analysis using the Protein Analysis Through Evolutionary Relationships (PANTHER) classification system [29,30]. The fold change in the normalized transcript counts was visualized with a heat map, where the color intensity indicated the magnitude of the change relative to the mean of the control samples. For most upregulated genes after sucrose treatment, GeneMANIA software (https://genemania.org/; accessed on 12 February 2024) was used to predict their functions and interactions with other genes in a network [31].

### 2.6. Flow Cytometry Analysis

Cell viability and ET efficiency were quantified with flow cytometry. For this analysis, 24 h after ET, the cells were detached from culture plates and washed twice with PBS. Collected samples were then stained with 5 μg/mL propidium iodide (PI) diluted in the flow buffer for the detection of dead cells, and the samples were fully vortexed. EGFP and PI signals were simultaneously detected in 488 nm and 633 nm channels, respectively, in the flow cytometer (Agilent Novocyte, Santa Clara, CA, USA). Data analysis was conducted using the Agilent Novocyte software (version 1.4.1) to quantify the ET efficiency and cell viability, as described in previous studies [24]. Briefly, effectiveness of electrotransfer (eTE) was defined as the percentage of live cells expressing EGFP (PI−/EGFP+). The expression level was defined as the geometric mean of EGFP fluorescence intensity per cell among the PI−/EGFP+ cells. Similar to the previous studies [24,32,33,34], the cell viability (%) was calculated as 100 times the ratio of live cell (PI−) numbers between the experimental and control samples. The effective expression level was defined as the product of the eTE, expression level, and cell viability, which is a measure of the overall expression level in a sample.

### 2.7. Statistical Analyses

Comparisons among multiple groups were performed using one-way analysis of variance (ANOVA) tests with Bonferroni corrections applied. For each pair of groups in multiple comparisons, a difference in the data was considered to be statistically significant if the corrected *p*-value was less than 0.05.

## 3. Results

### 3.1. Sucrose Induces Differential Expression of Genes in Mammalian Cells

**Figure 1:**
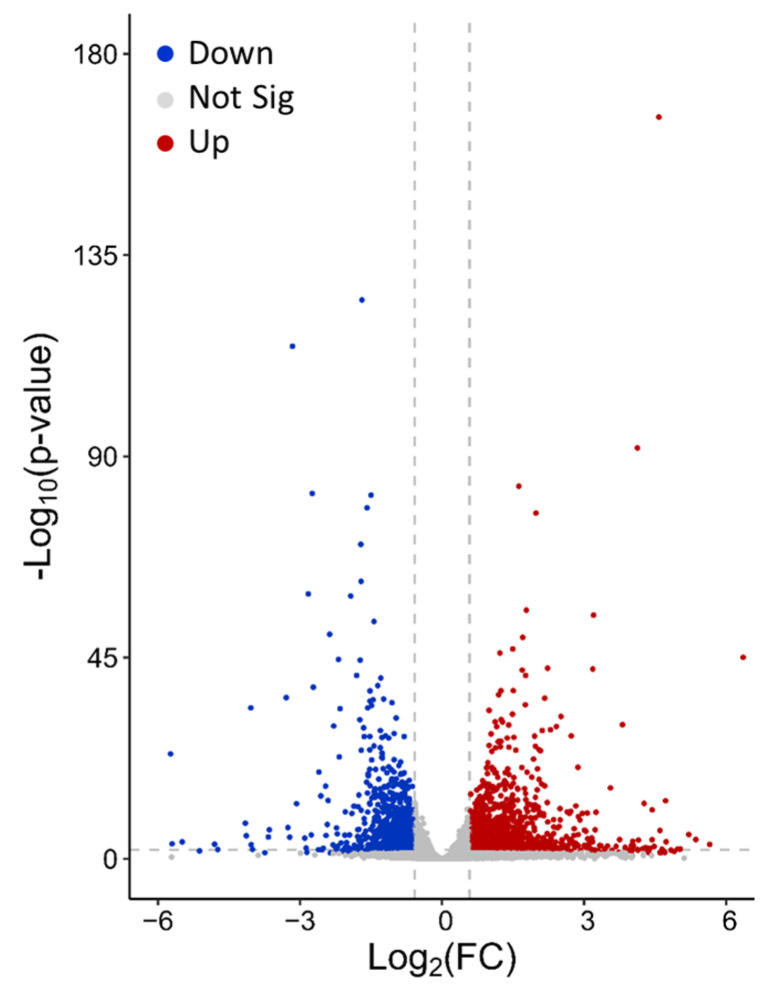
Differential gene expression induced through sucrose treatment. HCT116 cells were cultured for 24 h in a medium with or without a 100 mM sucrose supplement. Three biological repeats were prepared for the RNA sequencing analysis in both the sucrose-treated and non-treated groups; the sequence data were used to determine the average fold change (FC) and adjusted *p*-value for the differential expression of specific genes. Each point represents the data of a gene. The dashed lines represent −Log_10_(*p*-value) = 2, and Log_2_(FC) = ±0.58, respectively, and the adjusted *p*-value was used in the calculation. These lines divide the differential expression data into three categories. Gray points: non-significant (Not Sig) change wherein the magnitude of Log_2_(FC) is <0.58 or −Log_10_(*p*-value) is <2. Red points: upregulated (Up) transcripts wherein Log_2_(FC) is >0.58 and −Log_10_(*p*-value) is >2. Blue points: downregulated (Down) transcripts wherein Log_2_(FC) is <−0.58 and −Log_10_(*p*-value) is >2.

Transcriptomic profiles in the HCT116 cells were analyzed before and after the cells were cultured in the medium containing sucrose for 24 h. The sucrose concentration was maintained at 100 mM, an optimal value determined in our previous study [24]. Among the 16,242 transcripts detected in the RNA sequence analysis, 2198 showed significant a fold change (FC) ($\left|{Log}_{2}(FC)\right|$ > 0.58, adjusted *p*-value < 0.01), with 1181 transcripts being upregulated and 1017 transcripts being downregulated (Figure 1). The data indicated that the sucrose treatment could affect the expression of many different genes either directly or indirectly through interactions in gene networks. The most statistically significant differential expressions were observed in AQP3, ANK1, and INSIG1 for the upregulated genes, and SLC7A1, ASNS, and ARRDC4 for the downregulated genes.

The normalized counts were used in the gene set enrichment analysis (GSEA) for identifying the pathways affected by the sucrose treatment. After applying gene set filters to eliminate smaller and larger sets, 7604 gene sets annotated with the Gene Ontology (GO) terms were examined in the study. Among them, 4922 were overrepresented, with 1437 demonstrating a statistically significant overrepresentation (nominal *p*-value < 0.05 and false discovery rate (FDR) < 0.25). Moreover, 2682 were underrepresented, and the underrepresentation was statistically significant in 846 sets. The enrichment results were verified by using another approach to gene enrichment analysis (PANTHER). For both the overrepresented and underrepresented gene sets, their fold enrichments were larger than unity.

**Figure 2:**
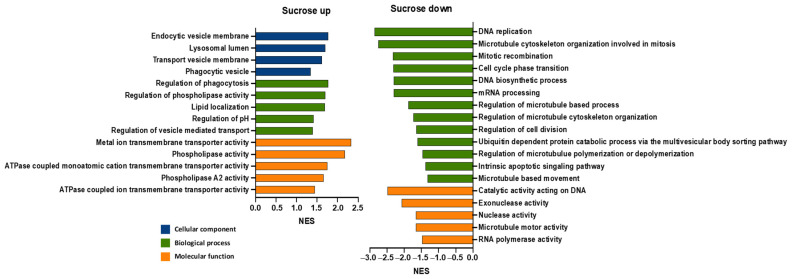
Enrichment of cellular pathways after sucrose treatment. Gene Ontology (GO) enrichment analysis was performed for normalized counts of transcripts in HCT116 cells treated with or without sucrose, based on three biological repeats in both groups, respectively. This plot only includes the enriched GO terms in three categories (cellular components, biological processes, and molecular functions) that were potentially linked to the enhancement of ET caused by sucrose treatment. NES, normalized enrichment score; Sucrose up, overrepresented GO terms with a positive NES; Sucrose down, underrepresented GO terms with a negative NES.

To understand how the sucrose treatment could affect ET, we focused on the GO terms that were potentially linked to the underlying biology of ET (Figure 2) [24]. Several significantly enriched or overrepresented GO terms were found to be associated with lipid membranes, which might explain our previous observations regarding the formation of large ALBs in mammalian cells after treatment with sucrose [24]; certain significantly underrepresented GO terms were associated with nucleic acid and protein catabolic pathways, which was consistent with our previous observations, wherein sucrose treatment decreased the degradation of plasmid DNA and reporter proteins in cells [24]. Furthermore, the data shown in Figure 2 indicate that the gene sets related to cell proliferation were underrepresented in the samples treated with sucrose, which was observed in the previous study as well [24].

### 3.2. Sucrose Activates Phospholipase A2 and V-ATPase in Mammalian Cells

**Figure 3:**
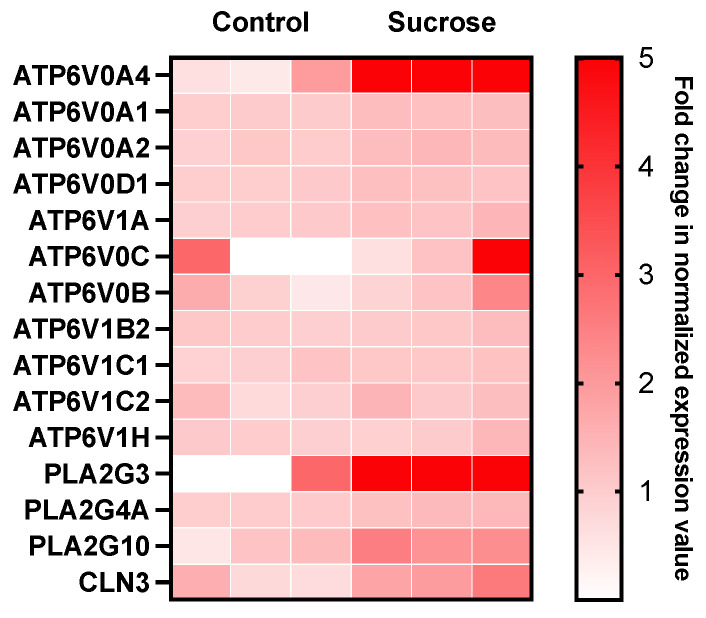
Heat map visualization of overexpressed genes. This map only includes the genes that were potentially linked to the enhancement of ET caused by sucrose treatment. They were identified through the analysis of differential gene expression and the gene set enrichment analysis. These analyses were based on three biological repeats in the control and sucrose-treated groups, respectively. The red color intensity indicates the magnitude of the fold change in the normalized transcript counts relative to the mean of the three control samples. The same intensity is assigned to magnitudes of five or higher.

Among the differentially expressed genes associated with the enriched pathways, we examined the upregulated genes related to ATPase-coupled cation transmembrane transporter activity, lipid localization, the endocytic vesicle membrane, phospholipase activity, and phospholipase A2 activity, which are potentially linked to the biological mechanisms of ET [24,35,36,37]. A list of these genes is shown in Figure 3, with the top two most significantly upregulated genes being ATP6V0A4 (up 6.7-fold) and PLA2G3 (up 13.5-fold).

**Figure 4:**
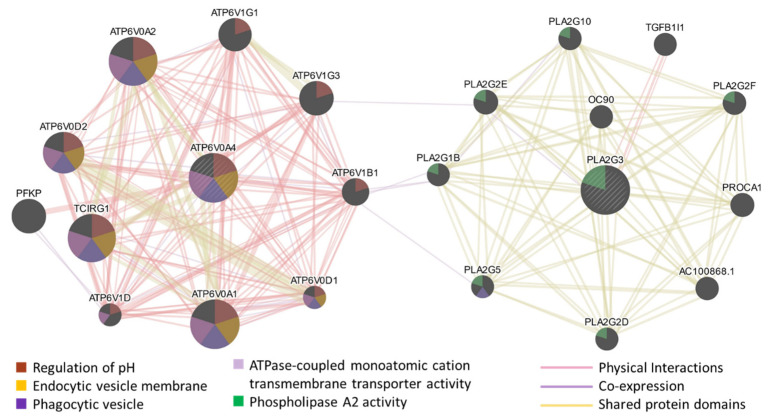
Gene network functionally associated with ATP6V0A4 and PLA2G3. This network was predicted by using the GeneMANIA algorithm. The prediction was influenced primarily by physical interactions, co-expression, and shared protein domains. The thickness of each line between two nodes indicates the extent of the predicted functional association between the two genes; the size of each node indicates the extent to which the predicted gene is related to ATP6V0A4 or PLA2G3. Functions of each gene were indicated by their association with five enriched GO terms potentially linked to the underlying biology of ET.

To further understand the functions of ATP6V0A4 and PLA2G3, a gene network functionally associated with these genes was predicted using the GeneMANIA algorithm (Figure 4) [31]. The network clearly showed two groups of genes. The V-ATPase-related genes were connected with ATP6V0A4, presumably because they share functions in pH regulation, transmembrane proton transport, and the vesicle membrane, whereas phospholipase A2-related genes were associated with PLA2G3 (Figure 4). The network prediction was influenced largely by the physical interactions and the co-expression of genes, with percent weights of 78% and 8%, respectively, and this influence appeared to be dominant in the first group. In the second group, the prediction was influenced primarily by the shared protein domains of the gene products. To show functions of the genes in the network, Figure 4 also includes information on how each gene was associated with five enriched GO terms potentially linked to the underlying biology of ET. These were the regulation of pH, the phagocytic vesicle, the endocytic vesicle membrane, ATPase-coupled monoatomic cation transmembrane transporter activity, and phospholipase A2 activity. Most genes in the first group were linked to the first four GO terms, while the genes in the second group were more closely linked to phospholipase A2 activity.

### 3.3. The Effects of Sucrose on ET Can Be Reversed via Inhibition of Phospholipase A2

The analysis above predicted that the sucrose treatment might enhance ET through the overexpression of genes associated with V-ATPase and secreted phospholipase A2. Thus, our next focus was to investigate whether the inhibition of V-ATPase or phospholipase A2 could reverse the effects of sucrose treatment on ET. Based on a literature review [38,39], we chose to use Concanamycin A (CMA) and Oleyloxyethyl Phosphorylcholine (OPC) as the inhibitors of V-ATPase and phospholipase A2, respectively. To determine the ranges of the inhibitor concentrations used in the treatments, we searched their IC_50_ values for inhibiting enzymatic activities. For OPC, the value provided by the manufacturer is 6.2 to 13.7 μM, depending on the source of the enzyme, and the IC_50_ value for CMA obtained from the literature is 10 nM [38]. However, previous data have shown that CMA at the IC_50_ value can induce significant apoptosis of cells [40]. To avoid this complication in the study, the cells were treated with CMA at relatively lower concentrations, causing the inhibition of V-ATPase activity to be less than 50%.

**Figure 5:**
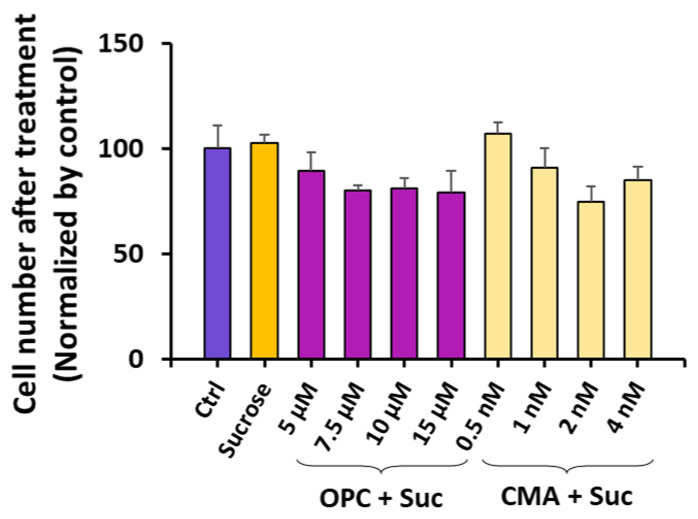
Number of viable cells after different treatments. HCT116 cells were cultured in medium supplemented with or without sucrose (100 mM) for 24 h. Then, the medium was aspirated and replaced with fresh medium containing no inhibitors (sucrose group) or different inhibitors at indicated concentrations (OPC + Suc and CMA + Suc groups). After the cells were cultured for another 6 h, the numbers of cells in individual groups were counted with a Countess II FL automated cell counter after trypan blue staining. The data were normalized according to the mean of the non-treated control group (Ctrl). The bar and error bar represent the mean and SD, respectively. Each group contains three independent repeats. No significant differences were observed between any treated groups and the Ctrl groups (corrected *p* > 0.05, one-way ANOVA).

When the cells were pretreated with 100 mM sucrose for 24 h, followed by a treatment with one of the inhibitors for 6 h at different concentrations (5 μM to 15 μM for OPC; 0.5 nM to 4 nM for CMA), the number of cells in each group was slightly lower than that in the non-treated control (Ctrl) (Figure 5). However, none of the differences between the treated and non-treated groups were statistically significant (corrected *p* > 0.05), suggesting that the treatments alone were minimally cytotoxic.

**Figure 6:**
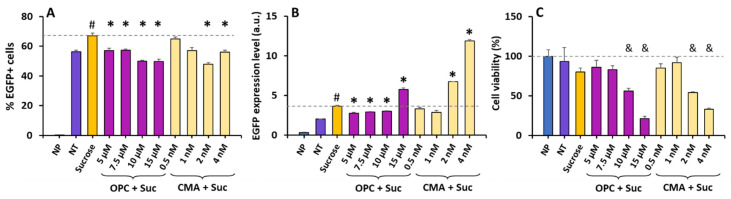
Effects of phospholipase A2 or V-ATPase inhibition on ET efficiency and cell viability. HCT116 cells were cultured in medium supplemented with or without sucrose (100 mM) for 24 h. Then, the medium was aspirated and replaced with fresh medium containing the indicated concentrations of inhibitors, and the cells were cultured for another 6 h prior to the electrotransfer of DNA encoding enhanced green fluorescence protein (EGFP). Twenty-four hours later, the cells were harvested for flow cytometry analysis to quantify (**A**) the %EGFP+ cells, (**B**) the EGFP expression level, and (**C**) the cell viability. The viability data were normalized according to the mean in the NP control group. The dashed lines in panels (**A**,**B**) represent the means in the sucrose group. The dashed line in panel (**C**) represents the mean in the NP group. NP, cells were not treated with sucrose or inhibitor, and no ET was performed; NT, ET was performed but the cells were not treated with sucrose or inhibitor. N = 3. #: corrected *p*-value < 0.05, sucrose group versus NT control; *: corrected *p*-value < 0.05, group treated with inhibitor and sucrose versus group treated with sucrose alone; &: corrected *p*-value < 0.05, any group versus NP control; a.u., arbitrary unit.

Next, we examined the effects of the treatments on ET efficiency and cell viability post ET. The cells were pretreated with sucrose for 24 h and one of the inhibitors for 6 h, as described above. The cells were washed with PBS, and pDNA encoding EGFP was electrotransferred into the cells. Compared to the non-treated (NT) control, the sucrose treatment alone increased the eTE (i.e., the percentage of EGFP+ cells) from 56% to 67% (Figure 6A), and the expression level of EGFP per live cell by 80% (Figure 6B). These increases in the eTE and the expression level could be reversed through subsequent treatment with OPC, except for the treatment at 15 µM, which led to a significant decrease in the eTE, which was consistent with the data in low-concentration groups, but an increase in the expression level (Figure 6A,B). This inconsistency was presumably caused by a significant cell (see the discussion below). The CMA treatment could also reverse the increase in the eTE at high concentrations (Figure 6A). However, it was ineffective in reversing the sucrose treatment-induced increase in the expression level at low concentrations (Figure 6B), and at high concentrations, it caused a further increase in the expression level (corrected *p* < 0.05).

The data shown in Figure 5 indicate that the treatments with sucrose alone or in combination with the inhibitors caused minimal cell death. The question at hand was whether combining the same treatments with ET would result in cytotoxicity. To answer this question, we measured the cell viability 24 h post ET, and compared the data between different groups. The comparison revealed that the ET alone with no treatment (NT) or the combination of ET and the sucrose treatment caused minimal cell death (corrected *p* > 0.05) (Figure 6C). However, the addition of the inhibitor treatment between the sucrose treatment and the ET exhibited concentration-dependent cytotoxicity, with statistically significant cell loss observed in the top two concentration groups for both inhibitors (corrected *p* < 0.05) (Figure 6C).

**Figure 7:**
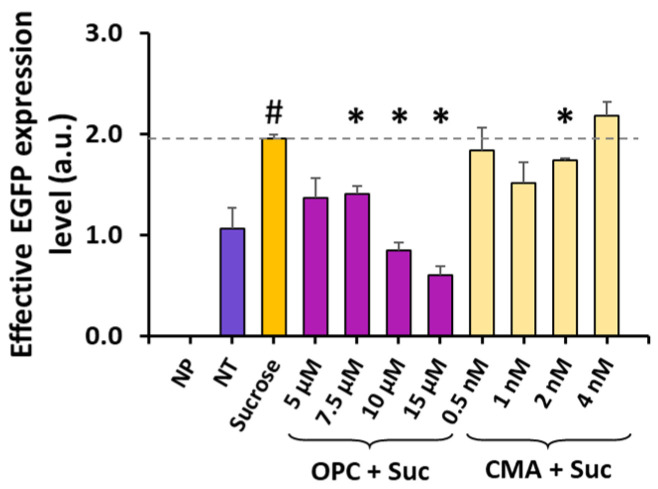
Effects of phospholipase A2 or V-ATPase inhibition on effective expression level. The data shown in Figure 6 were combined together to demonstrate the effects of the inhibitor treatments on the total amount of EGFP per sample. N = 3. The dashed line represents the means in the sucrose group. NP, cells were not treated with sucrose nor inhibitor, and no ET was performed; NT, ET was performed but the cells were not treated with sucrose or inhibitor. N = 3. #: corrected *p*-value < 0.05, sucrose group versus NT control group; *: corrected *p*-value < 0.05, group treated with inhibitor and sucrose versus group treated with sucrose alone; a.u., arbitrary unit.

The cell death reduced transgene protein (i.e., EGFP) production in the samples. The total amount of the protein divided by the number of cells prior to the treatment and the ET was defined as the effective expression level per cell. Compared to the NT control, the sucrose treatment alone approximately doubled the effective expression level (corrected *p* < 0.05) (Figure 7). This increase could be reversed through subsequent treatment with OPC in a concentration-dependent manner (Figure 7). However, the CMA treatment was ineffective at reversing the increase in the effective expression level induced by the sucrose treatment (Figure 7). The data demonstrated that the enhancement in ET efficiency induced by the sucrose treatment was mediated primarily by activating phospholipase A2.

## 4. Discussion

Our previous studies demonstrated that sucrose treatment can enhance ET efficiency and that this enhancement is achieved through changes in vesicle functions and vesicle–vesicle interactions in cells [24,26]. To understand the molecular mechanisms of these changes, the current study compared transcriptomic profiles in sucrose-treated and non-treated cells based on RNA-seq analysis, a powerful technique for identifying differentially expressed genes [41]. Furthermore, data from the analysis were utilized to determine the enrichment of pathways and gene sets induced by the sucrose treatment. The results from this study reveal that the sucrose treatment resulted in the underrepresentation of gene sets linked to nucleic acid and protein degradation as well as cell cycle regulation, indicating that the treatment downregulated the genes involved in these processes. Meanwhile, the treatment led to the enrichment or overrepresentation of gene sets associated with phospholipase activity, lipid localization, and ATPase-coupled ion transmembrane transporter activity, suggesting that the treatment upregulated the genes involved in vesicle trafficking, lipid metabolism, and the transmembrane transport of ions in cells. Specifically, we observed a significant overexpression of two genes (ATP6V0A4 and PLA2G3) in the enriched gene sets, which are associated with V-ATPase and secreted phospholipase A2, respectively. Inhibiting the phospholipase A2 activity with a pharmaceutical inhibitor (OPC) could reverse the increase in ET efficiency elicited by the sucrose treatment. However, treatment of the cells with CMA, a pharmaceutical inhibitor of V-ATPase, was ineffective at reversing the effects of the sucrose on ET. The data suggest that the sucrose treatment enhances ET efficiency primarily through the activation of phospholipase A2.

Phospholipase A2 plays an important role in vesicle trafficking [42]. It is involved in the regulation of lipid membrane curvature through local hydrolysis of membrane phospholipids at the sn-2 position. This enzymatic activity produces metabolites that introduce spontaneous curvature stress to the membrane, influencing the bending of the membrane locally via alteration of the lipid packing and the conformation of membrane proteins [42]. As a result, it can influence membrane deformation, budding, and fusion processes. Data in the current study demonstrated that PLA2G3 was overexpressed in cells treated with sucrose, upregulating phospholipase A2 activity. This upregulation potentially drives vesicle fusion to form large multi-origin vesicles (e.g., ALBs), such as those observed in our previous study [24]. The increased vesicle fusion could also contribute to decreased lysosomal acidity, as the fusion between lysosomes and other non-acidic vesicles would dilute the protons in lysosomes, reducing its acidity [24].

The V-ATPase complex is responsible for acidifying vesicles in eukaryotic cells through facilitating proton transport across vesicular membranes [43,44]. Data from the current study demonstrated that the sucrose treatment upregulated the expression of the gene (ATP6V0A4) encoding the α4 subunit of V-ATPase [45], implying that the treatment increased the V-ATPase activity in treated cells. This activity increase is expected to cause an increase in the acidity of vesicles, including late endosomes and lysosomes, which appears to contradict our previous observations of sucrose treatment decreasing the number of acidic lysosomes [24,26]. To elucidate this discrepancy, we hypothesize that the sucrose treatment decreases the lysosomal acidity through mechanisms associated with vesicle fusion, independent of the V-ATPase activity. This hypothesis is supported by two observations in the current study. First, the sucrose treatment could enhance vesicle fusion through the upregulation of phospholipase A2 activity, as discussed above. The fusion between non-acidic vesicles and lysosomes caused a decrease in lysosomal acidity. Second, the CMA treatment was ineffective at reversing the increase in ET efficiency induced by the sucrose treatment (Figure 6 and Figure 7). And, at higher concentrations, the CMA treatment could further augment the effects of the sucrose treatment on ET, presumably due to a decrease in the influx of protons into the lysosome. It is also worth mentioning that some genes strongly associated with ATP6V0A4, as predicted with GeneMANIA, were not overexpressed in the sucrose-treated cells (Figure 3 and Figure 4), indicating that these associated genes are not crucial for the ET enhancement. The observations discussed above suggest that this sucrose treatment activates both phospholipase A2 and V-ATPase, and that any potential decrease in the lysosomal pH caused by V-ATPase activation can be offset via the activation of phospholipase A2. As a result, the lysosomal pH was increased, which led to inactivation of the nucleases and a subsequent decrease in the degradation of electrotransferred DNA within the lysosome [24,26]. Additional studies are needed to further investigate this hypothesis.

In summary, the findings in this study reveal the molecular targets of sucrose treatment in mammalian cells that can be exploited for improving gene delivery based on ET. The data in this study explain how the sucrose treatment was able to increase the efficiency of ET through interfering with vesicle trafficking and fusion, which led to a decrease in lysosomal degradation of the exogenous DNA endocytosed by cells. These data underscore the potential of using sucrose as a nontoxic and non-inflammatory agent to improve the outcomes of ET in clinical applications.

## Data Availability

All data needed to support the conclusions are present in the paper. Raw data are available from the corresponding author, F.Y., upon reasonable request.
